# Serological Evidence of Filovirus Infection in Nonhuman Primates in Zambia

**DOI:** 10.3390/v13071283

**Published:** 2021-06-30

**Authors:** Katendi Changula, Edgar Simulundu, Boniface Pongombo Lombe, Eri Nakayama, Hiroko Miyamoto, Yuji Takahashi, Hirofumi Sawa, Chuma Simukonda, Bernard M. Hang’ombe, Ayato Takada

**Affiliations:** 1 Department of Paraclinical Studies, School of Veterinary Medicine, University of Zambia, Lusaka 10101, Zambia; katendi.changula@sacids.org (K.C.); mudenda68@yahoo.com (B.M.H.); 2 Department of Disease Control, School of Veterinary Medicine, University of Zambia, Lusaka 10101, Zambia; esikabala@yahoo.com (E.S.); h-sawa@czc.hokudai.ac.jp (H.S.); 3 Macha Research Trust, P.O. Box 630166, Choma 20100, Zambia; 4 Division of Global Epidemiology, International Institute for Zoonosis Control, Hokkaido University, Sapporo 001-0020, Japan; bonifacelombe@czc.hokudai.ac.jp (B.P.L.); hirom@czc.hokudai.ac.jp (H.M.); y-takahashi@czc.hokudai.ac.jp (Y.T.); 5 Central Veterinary Laboratory of Kinshasa, Kinshasa BP 8842, Democratic Republic of the Congo; 6 Faculty of Veterinary Medicine, National Pedagogic University, Kinshasa BP 8815, Democratic Republic of the Congo; 7 Department of Virology I, National Institute of Infectious Diseases, Tokyo 162-0052, Japan; nakayama@nih.go.jp; 8 Division of Molecular Pathobiology, International Institute for Zoonosis Control, Hokkaido University, Sapporo 001-0020, Japan; 9 International Collaboration Unit, International Institute for Zoonosis Control, Hokkaido University, Sapporo 001-0020, Japan; 10 Department of National Parks and Wildlife, Chilanga 10101, Zambia; chumasimukonda@gmail.com

**Keywords:** Ebola virus, ebolavirus, Marburg virus, marburgvirus, filovirus, nonhuman primate, antibody, seroprevalence, Zambia

## Abstract

Ebolaviruses and marburgviruses are filoviruses that are known to cause severe hemorrhagic fever in humans and nonhuman primates (NHPs). While some bat species are suspected to be natural reservoirs of these filoviruses, wild NHPs often act as intermediate hosts for viral transmission to humans. Using an enzyme-linked immunosorbent assay, we screened two NHP species, wild baboons and vervet monkeys captured in Zambia, for their serum IgG antibodies specific to the envelope glycoproteins of filoviruses. From 243 samples tested, 39 NHPs (16%) were found to be seropositive either for ebolaviruses or marburgviruses with endpoint antibody titers ranging from 100 to 25,600. Interestingly, antibodies reactive to Reston virus, which is found only in Asia, were detected in both NHP species. There was a significant difference in the seropositivity for the marburgvirus antigen between the two NHP species, with baboons having a higher positive rate. These results suggest that wild NHPs in Zambia might be nonlethally exposed to these filoviruses, and this emphasizes the need for continuous monitoring of filovirus infection in wild animals to better understand the ecology of filoviruses and to assess potential risks of outbreaks in humans in previously nonendemic countries.

## 1. Introduction

Filoviruses, including ebolaviruses and marburgviruses, have a non-segmented negative-stranded RNA genome and are phylogenetically divided into six genera: *Ebolavirus*, *Marburgvirus*, *Cuevavirus*, *Dianlovirus*, *Striavirus*, and *Thamnovirus*. The genus *Marburgvirus* has a single species with two known viruses (Marburg virus (MARV) and Ravn virus), but six distinct species are currently known in the genus *Ebolavirus*: *Zaire ebolavirus*, *Sudan ebolavirus*, *Tai Forest ebolavirus*, *Bundibugyo ebolavirus*, *Reston ebolavirus*, and *Bombali ebolavirus*, represented by Ebola virus (EBOV), Sudan virus (SUDV), Taï Forest virus (TAFV), Bundibugyo virus (BDBV), Reston virus (RESTV), and Bombali virus, respectively [1]. Of these filoviruses, MARV, EBOV, SUDV, TAFV, and BDBV are known to be human-pathogenic filoviruses that cause hemorrhagic fever, namely Marburg virus disease (MVD) and Ebola virus disease (EVD) in humans and nonhuman primates (NHPs) [2]. RESTV causes disease in NHPs and has also been found in pigs [3,4]. Although it is not known to cause disease in humans, there have been reports of seroconversion in humans after contact with RESTV-infected NHPs or pigs [3,4].

NHPs are known to be highly susceptible to filovirus infection. In some of the previous outbreaks of EVD, large die-offs of NHPs have been recorded prior to or during the outbreaks [5,6,7,8]. In addition, NHPs have been implicated as a source of human infection in some outbreaks, with the index cases having handled infected NHP carcasses [7,9]. The source of infection in the first ever recorded outbreak of a filovirus disease, MVD, was African green monkeys [10]. Antibodies to various filoviruses have been detected in several NHP species, both in areas of previously reported outbreaks and those that had never reported an outbreak [11,12,13,14,15,16].

The aim of this study was to investigate the possible exposure of NHPs to filoviruses in Zambia, where neither an EVD nor an MVD outbreak has ever been recorded. We screened 243 serum samples collected from NHPs in Zambia for the detection of filovirus-specific IgG. There was evidence of previous exposure to filoviruses in these NHP populations.

## 2. Materials and Methods

### 2.1. Animals and Serum Samples

**Figure 1:**
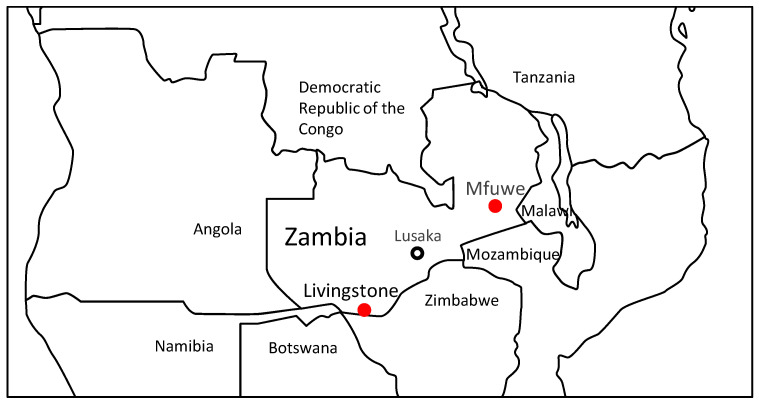
Map of Zambia showing the location of the sampling sites (Mfuwe and Livingstone).

Serum samples were previously obtained from 125 (41 female and 84 male) yellow baboons (*Papio cynocephalus*) and 118 (33 female and 85 male) vervet monkeys (*Chlorocebus pygerythrus*) in game management areas in the Mfuwe region (Eastern Province, Zambia) and Livingstone (Southern Province, Zambia) (Figure 1) between 2008 and 2010 with the permission (certificate no. 2604) of the Zambia Wildlife Authority, which is mandated by the Zambian government to control the large numbers of wild NHPs [17,18,19]. Serum samples were stored at −80 °C until they were used. The residual serum samples were used in the present study.

### 2.2. Enzyme-Linked Immunosorbent Assay (ELISA)

Filovirus GP-based ELISA was performed as described previously [20,21,22]. Histidine-tagged soluble recombinant GPs of EBOV (Yambuku), SUDV (Nzara), TAFV (Pauléoula), BDBV (Butalya), RESTV (Philippines89), and MARV (Angola) were purified from the supernatants of human embryonic kidney 293T cells transfected with the pCAGGS plasmid expressing each GP by using the Ni-NTA Purification System (Invitrogen, CA, USA). Purified GP antigens were analyzed by sodium dodecyl sulfate-polyacrylamide gel electrophoresis and Western blotting, and were confirmed by prominent protein bands of the predicted size of GPs. ELISA plates (Maxisorp, Nunc, Thermo Fisher Scientific, Waltham, MA, USA) were coated with each purified GP antigen (1 µg/mL in phosphate-buffered saline, 50 µL/well) or a negative control antigen (FCS-derived proteins nonspecifically bound to Ni-beads) [20] overnight at 4 °C. This was followed by blocking with 3% skim milk, and serum samples (diluted at 1:1000 for initial screening and 4-fold serial dilutions from 1:400 for subsequent titration) were added. Bound antibodies were visualized with peroxidase-conjugated goat anti-monkey IgG (Rockland, Limerick, PA, USA) and 3,3′,5,5′-tetramethylbenzidine (Sigma, St. Louis, MO, USA). The reaction was stopped by adding 1N phosphoric acid, and the optical density (OD) at 450 nm was measured. To offset the nonspecific antibody reaction, the OD value of the control antigen was subtracted from the OD value of each sample. Assays were conducted in duplicate and averages were used for further data analyses. To specify a viral antigen to which each serum sample predominantly reacts, we concurrently tested all the antigens in parallel. We previously confirmed that the sensitivity of our assay did not vary among the GP antigens using control antisera for the respective viruses [20]. It is also important to note that, in our assay, if a serum sample shows a positive reaction specifically to one virus species, all the other antigens should work as negative control antigens. Thus, we used the whole population of the OD values for the statistical analysis described below.

### 2.3. Reverse Transcription Polymerase Chain Reaction (RT-PCR)

Total RNA was extracted from serum samples of individual animals using a QIAamp Viral RNA Mini Kit (QIAGEN, Hilden, Germany) according to the manufacturer’s instructions. One-step RT-PCR assays were carried out using a QIAGEN OneStep RT-PCR kit (QIAGEN) according to the manufacturer’s instructions using filovirus-specific universal primer sets targeting the NP gene, FiloNP-Fm, FiloNP-Rm, FiloNP-Fe, and FiloNP-Re as described previously [22].

### 2.4. Statistics

To determine the statistical significance of each OD value, the Smirnov–Grubbs rejection test, which is widely used to detect significantly higher or lower values (i.e., outliers) that do not belong to the population consisting of all other values in the data set, was employed as described previously [16,21]. Briefly, the highest OD value was first selected, and the statistical significance of the T value was calculated based on the critical values given by the Smirnov–Grubbs test. If it was considered to be an outlier, the T-value of the second highest OD value was similarly tested without the highest one. These steps were repeated until the T value fell to below the level of statistical significance (*p* < 0.05) and the cutoff OD values were determined. For comparison of seropositivity between NHP species, the chi-square test was used.

## 3. Results and Discussion

### 3.1. Serological and Genetic Screening of NHPs for Filoviruses

**Figure 2:**
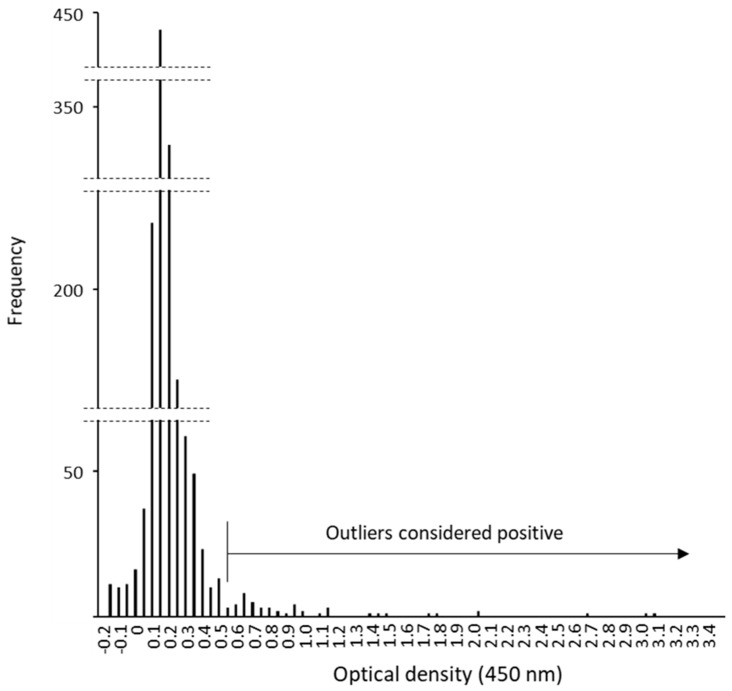
The frequency distribution of the NHP serum samples according to OD values obtained by GP-based ELISA. Serum samples were tested (1:1000 dilution) for IgG antibodies specific to EBOV, SUDV, TAFV, BDBV, RESTV, and MARV GPs. All OD values (6 virus species for 243 samples, *n* = 1458) were subjected to the Smirnov–Grubbs rejection test to discriminate the positive (i.e., significantly higher outlier values) from the negative population. The frequency distribution chart reveals that the sample population consists of a major single peak with low OD values (approximately < 0.5) and outliers (*p* < 0.05) with high OD values (approximately > 0.55).

ELISA was used to screen NHP serum samples for IgG antibodies to filovirus GPs. As there were no baboon and vervet monkey sera available for positive and negative controls, the Smirnov–Grubbs rejection test was used to determine the statistical significance of the OD values obtained. Outliers with high OD values of approximately > 0.55 were considered to be positive (Figure 2).

Of the 243 NHP serum samples that were screened, 39 (16%) tested positive for filovirus IgG (Table 1). However, no filoviral RNA genomes were detected in this study. It has been reported that acute filovirus infection is cleared from both naturally and experimentally infected NHPs within three weeks of infection, and IgG antibodies following the infection generally persist for years [23,24]. These observations imply that the NHPs were infected in the past but had no active infection at the time of sampling.

### 3.2. Filoviruses-Species Specificity of Serum IgG Antibodies Detected in NHPs

**Figure 3:**
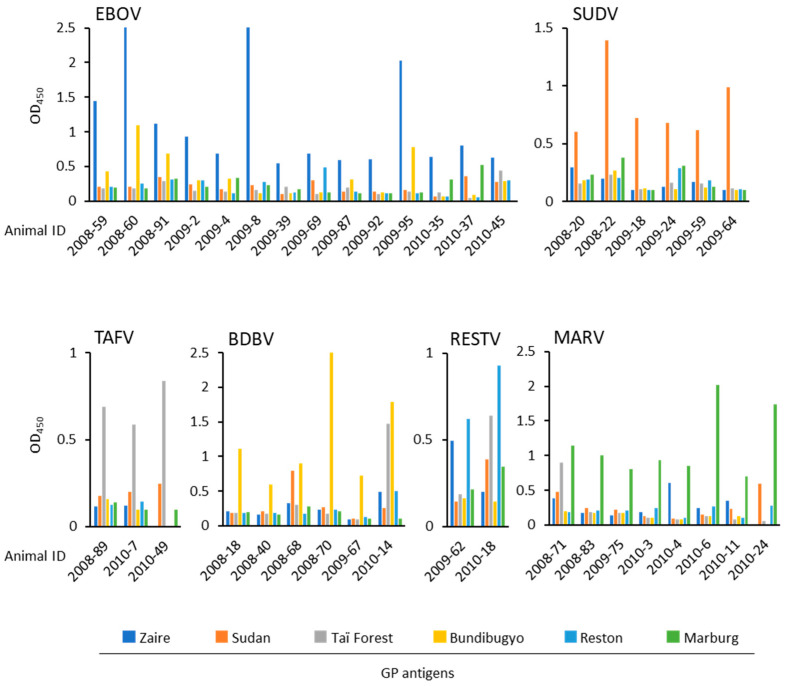
Filovirus species-specificity of IgG antibodies detected in the sera collected from NHPs in Zambia. IgG reactivities to EBOV, SUDV, TAFV, BDBV, RESTV, and MARV GPs are shown as OD values at 450 nm (OD450) in ELISA for the 39 positive samples shown in Table 1.

IgG antibodies to filoviruses that are pathogenic to humans (EBOV, SUDV, TAFV, BDBV, and MARV), as well as RESTV, which is not known to cause human disease, were detected throughout all years of sampling, apart from RESTV in 2008, TAFV in 2009, and SUDV in 2010 (Table 1, Figure 3). Overall, seropositivity to ebolavirus species was 12.8% (31/243), with the highest seropositivity to EBOV (5.8%, 14/243) and the lowest to RESTV (0.8%, 2/243), whereas the seropositivity to MARV was 3.3% (8/243). The end point titers of samples testing positive ranged from 1:400 to 1:25,600 (Table 2). There was no significant difference in the positive rates between female and male NHPs (data not shown).

Some of the positive samples showed cross-reactivity (i.e., above the cutoff OD value) to GPs of multiple filovirus species (Figure 3). For instance, antibodies reactive to BDBV were also detected in three of the EBOV IgG-positive sera (#2008-60, #2008-91, and #2009-95). Since the phylogenetical relationship between EBOV and BDBV is relatively close and IgG antibodies in EBOV-infected human sera reacted to BDBV [20], this may be explained as typical cross-reactivity of anti-EBOV sera to BDBV. Similar cross-reactivity was seen between TAFV and BDBV in one of the BDBV-positive sera (#2010-14). On the other hand, one of the BDBV-positive sera (#2008-68), two RESTV-positive sera (#2009-62 and #2010-18), and three of the MARV-positive sera (#2008-71, #2010-4, and #2010-24) showed reactivity to phylogenetically and antigenically distant filoviruses. We speculate that these NHPs were infected with an antigenically distinct unknown filovirus or infected independently with different filoviruses.

The evidence of exposure of NHPs to EBOV, MARV, and BDBV is of note since all these viruses have caused outbreaks in countries neighboring Zambia [25,26]. The seropositivity rate of EBOV in this study (5.8%) was higher than that found in NHPs in previous studies in areas that have recorded outbreaks of EVD. A study of NHPs in Gabon and the Republic of the Congo reported EBOV seropositivity rates of 1.1% and 1.4% (baboons, chimpanzees, gorillas, and others in the genera *Mandrillus* and *Cercopithecus*), respectively [11]. Another study on NHPs from Cameroon, the Democratic Republic of the Congo, and Ivory Coast had an overall seroprevalence to EBOV IgG of 2.6% (NHP species in the genera *Cercocebus*, *Colobus*, *Cercopithecus*, and *Chlorocebus*) [14]. However, results between these studies and ours may not be comparable as the target antigen was not described [11] and a different assay with different cut off values was used [14]. In addition, our higher prevalence could also have been caused by increased sensitivity due to the use of a secondary anti-monkey antibody instead of a secondary anti-human antibody in the ELISA.

In addition, some NHPs tested in our study were seropositive for TAFV and SUDV, whose known distributions are West Africa and East Africa, respectively [5,27]. Interestingly, antibodies reactive to RESTV, which is thought to be an Asian filovirus, were also detected in two NHPs. We previously detected RESTV-specific IgG in fruit bats in Zambia [21,28]. This may indicate that RESTV or RESTV-like filoviruses are circulating in Africa, including Zambia. While RESTV is known to be highly pathogenic to some NHP species, the putative RESTV-like virus may be less lethal, either due to reduced pathogenicity of the virus or resistance of some animals to the RESTV-like virus, as has been seen in African green monkeys [29]. Similarly, IgG antibodies reactive to EBOV, SUDV, TAFV, BDBV, and MARV may not be conclusive evidence of exposure to these previously known filoviruses since it is also conceivable that the detected antibodies might be induced by infection with as yet undiscovered filoviruses, whose antigenicities are related to the respective known viruses but have reduced pathogenicity.

The relatively high prevalences of EBOV, SUDV, BDBV, and MARV were surprising considering that no outbreak of EVD/MVD has ever been reported in Zambia, and has implications for the possibility of an outbreak in these NHP species and potential spillover to humans. Spillover events may occur when baboons and vervet monkeys, which are considered to be pests, raid farmers’ crops, approach human dwellings for food [30,31,32,33], or when they are kept as pets [34]. Other studies have also found evidence of exposure of both humans and NHPs to filoviruses in areas where outbreaks have not been recorded [11,16,35,36,37]. This information suggests that sporadic occurrences of filovirus infection may be largely unrecognized since EVD and MVD show similar symptoms to other tropical diseases, such as malaria and gastroenteritis [38,39], and therefore are not a part of the routine diagnostic workup. Bush meat (e.g., NHPs and bats) consumption, including illegal bush meat trades, may also be a key factor that influences the frequency of spillover to humans. Our results suggest that there may be a wider geographic distribution of EBOV, SUDV, TAFV, BDBV, RESTV, and MARV than has been previously assumed, and/or circulation of filoviruses that are yet to be discovered, which could be antigenically similar to, but less pathogenic, than these known filoviruses. 

During our study period, the serologically predominant filoviruses were EBOV, SUDV, BDBV, and MARV (Table 1). The relatively higher seropositive rates of the NHPs tested in the present study may indicate an increased risk of spillover of these human-pathogenic filoviruses to humans. A correlation between predominant seropositivity and filovirus species actually causing the outbreaks in humans was suggested in our previous study on migratory fruit bats [21]. However, there is no evidence that the NHP populations in this study were migratory, and it has been shown that monkeys and baboons tend to stay within a specific home range [40,41].

### 3.3. Difference in the Seropositivity for MARV between Baboons and Vervet Monkeys

The seropositivities of baboons and vervet monkeys were comparable for all ebolaviruses tested in this study, with the highest positive rate for EBOV in both NHP species, followed by SUDV and BDBV, TAFV, and RESTV (Table 3). In contrast, there was a significant difference in the MARV seropositivity rates between baboons (5.6%, 7/125) and vervet monkeys (0.8 %, 1/118). Higher seropositivity implies increased exposure to the virus. As these NHPs were sampled from the same area and generally ate the same food, there should be other factors contributing to this difference and this is worth further investigation. 

We recently reported a high seroprevalence of antibodies to MARV, as well as the detection of the MARV genome, in Egyptian fruit bats in Zambia, indicating that marburgviruses are circulating in these cave-dwelling bat species in Zambia [28,42]. Baboons prefer sleeping in caves when they are available [43], which could increase their risk of MARV exposure. In addition, baboons are a predator of vervet monkeys, and one study that investigated the foraging habits of baboons and vervet monkeys on a commercial farm at the edge of a forest observed that vervet monkeys retreated from the area when baboons were foraging, which was mainly in the morning [31]. This may suggest that baboons have an increased probability of exposure to MARV after handling and eating contaminated fruits discarded by infected Egyptian fruit bats during the night [44]. In addition, baboons have been observed eating bats, which increases the risk of infection with viruses [45]. Although direct evidence for the presence of caves and colonies of Egyptian fruit bats in the study areas is lacking, this bat species may be widespread in the Eastern and Southern African region including Zambia [46]. The ecology, social behavior, and foraging habits of NHPs in relation to their risk for zoonotic infections thus require further exploration.

## 4. Conclusions

This study assessed the filovirus exposure of baboons and vervet monkeys in Zambia. Despite no outbreak of EVD or MVD ever being reported in Zambia, our data provide serological evidence of filovirus infection in these NHPs. In both NHP species, the seroprevalence to EBOV was the highest, though antibodies to other filoviruses were also detected. Baboons had a significantly higher seroprevalence to MARV than vervet monkeys. The evidence of filovirus infection in NHPs, which are considered susceptible hosts, indicates that they are in contact with the reservoir and/or amplifying hosts. The results of this study, taken together with our previous studies conducted in Zambia, suggest that filoviruses are circulating in some wild animal species in the country. Thus, there is a need for surveillance of filoviruses in both human and wildlife populations in Zambia to further understand the epidemiology of filovirus infection.

## Figures and Tables

**Table 1:** Seropositive rates of NHPs for filoviruses.

Year	% (Positive/Total) for Each Filovirus ^1^						
	EBOV	SUDV	TAFV	BDBV	RESTV	MARV	Total
2008	3.2	2.1	1.1	4.3	0	2.1	12.8
	(3/94)	(2/94)	(1/94)	(4/94)	(0/94)	(2/94)	(12/94)
2009	8.1	4.0	0	1.0	1.0	1.0	15.2
	(8/99)	(4/99)	(0/99)	(1/99)	(1/99)	(1/99)	(15/99)
2010	6.0	0	4.0	2.0	2.0	10.0	24.0
	(3/50)	(0/50)	(2/50)	(1/50)	(1/50)	(5/50)	(12/50)
Total	5.8	2.5	1.2	2.5	0.8	3.3	16.0
	(14/243)	(6/243)	(3/243)	(6/243)	(2/243)	(8/243)	(39/243)

^1^ The filovirus species for which each EBOV-positive sample had the highest OD value in the GP-based ELISA was selected when a sample showed cross-reactivity to GPs of multiple species.

**Table 2:** Distribution of antibody titers of the IgG positive sera.

Antigen	Antibody Titer ^1^				Total
	400	1600	6400	25,600	
EBOV	3	7	3	1	14
SUDV	2	4	0	0	6
TAFV	0	3	0	0	3
BDBV	1	3	1	1	6
RESTV	1	0	1	0	2
MARV	3	4	1	0	8

**^1^** Titers were expressed as the reciprocal of the highest dilution which gave an OD value above background.

**Table 3:** Seropositive rates of baboons and vervet monkeys to each filovirus species.

NHP Species	% (Positive/Total) for Each Filovirus ^1^					
	EBOV	SUDV	TAFV	BDBV	RESTV	MARV ^2^
Baboon	6.4	2.4	0.8	1.6	0.8	5.6
	(8/125)	(3/125)	(1/125)	(2/125)	(1/125)	(7/125)
Vervet monkey	5.1	2.5	1.7	3.4	0.8	0.81
	(6/118)	(3/118)	(2/118)	(4/118)	(1/118)	(1/118)
Total	5.8	2.5	1.2	2.5	0.8	3.3
	(14/243)	(6/243)	(3/243)	(6/243)	(2/243)	(8/243)

^1^ Same as Table 1. ^2^ A significant difference was found between baboons and vervet monkeys (*p* < 0.05).

## Data Availability

The data presented in this study are available on request from the corresponding author.

## References

[1] Kuhn J.H., Adkins S., Alioto D., Alkhovsky S.V., Amarasinghe G.K., Anthony S.J., Avšič-Županc T., Ayllón M.A., Bahl J., Balkema-Buschmann A. (2020). 2020 taxonomic update for phylum Negarnaviricota (Riboviria: Orthornavirae), including the large orders Bunyavirales and Mononegavirales. Arch. Virol..

[2] Feldmann H., Sanchez A., Geisbert T.W., Knipe D.M., Howley P.M., Cohen J.I., Griffin D.E., Lamb R.A., Martin M.A., Racaniello V.R., Roizman B. (2013). Filoviridae: Marburg and Ebola viruses. Fields Virology.

[3] Barrette R.W., Metwally S.A., Rowland J.M., Xu L., Zaki S.R., Nichol S.T., Rollin P.E., Towner J.S., Shieh W.J., Batten B. (2009). Discovery of swine as a host for the Reston ebolavirus. Science.

[4] Jahrling P.B., Geisbert T.W., Dalgard D.W., Johnson E.D., Ksiazek T.G., Hall W.C., Peters C.J. (1990). Preliminary report: Isolation of Ebola virus from monkeys imported to USA. Lancet.

[5] Formenty P., Boesch C., Wyers M., Steiner C., Donati F., Dind F., Walker F., Le G.B. (1999). Ebola virus outbreak among wild chimpanzees living in a rain forest of Cote d’Ivoire. J. Infect. Dis..

[6] Pourrut X., Kumulungui B., Wittmann T., Moussavou G., Delicat A., Yaba P., Nkoghe D., Gonzalez J.P., Leroy E.M. (2005). The natural history of Ebola virus in Africa. Microbes. Infect..

[7] Leroy E.M., Rouquet P., Formenty P., Souquiere S., Kilbourne A., Froment J.M., Bermejo M., Smit S., Karesh W., Swanepoel R. (2004). Multiple Ebola virus transmission events and rapid decline of central African wildlife. Science.

[8] Bermejo M., Rodriguez-Teijeiro J.D., Illera G., Barroso A., Vila C., Walsh P.D. (2006). Ebola outbreak killed 5000 gorillas. Science.

[9] Le Guenno B., Formenty P., Wyers M., Gounon P., Walker F., Boesch C. (1995). Isolation and partial characterisation of a new strain of Ebola virus. Lancet.

[10] Slenczka W. (2017). Filovirus Research: How it Began. Curr. Top Microbiol. Immunol..

[11] Leroy E.M., Telfer P., Kumulungui B., Yaba P., Rouquet P., Roques P., Gonzalez J.P., Ksiazek T.G., Rollin P.E., Nerrienet E. (2004). A serological survey of Ebola virus infection in central African nonhuman primates. J. Infect. Dis..

[12] Johnson B.K., Gitau L.G., Gichogo A., Tukei P.M., Else J.G., Suleman M.A., Kimani R., Sayer P.D. (1982). Marburg, Ebola and Rift Valley Fever virus antibodies in East African primates. Trans. R. Soc. Trop. Med. Hyg..

[13] Reed P.E., Mulangu S., Cameron K.N., Ondzie A.U., Joly D., Bermejo M., Rouquet P., Fabozzi G., Bailey M., Shen Z. (2014). A new approach for monitoring ebolavirus in wild great apes. PLoS Negl. Trop. Dis..

[14] Ayouba A., Ahuka-Mundeke S., Butel C., Mbala K.P., Loul S., Tagg N., Villabona-Arenas C.J., Lacroix A., Ndimbo-Kumugo S.P., Keita A.K. (2019). Extensive serological survey of multiple African nonhuman primate species reveals low prevalence of immunoglobulin G antibodies to 4 Ebola virus species. J. Infect. Dis..

[15] Ogunro B.N., Olugasa B.O., Verschoor E.J., Olarinmoye A.O., Theyse I., Niphuis H. (2018). Serological detection of Ebola virus exposures in native non-human primates of southern Nigeria. J. Epidemiol. Glob. Health.

[16] Nidom C.A., Nakayama E., Nidom R.V., Alamudi M.Y., Daulay S., Dharmayanti I.N., Dachlan Y.P., Amin M., Igarashi M., Miyamoto H. (2012). Serological evidence of Ebola virus infection in Indonesian orangutans. PLoS ONE.

[17] Sasaki M., Ishii A., Orba Y., Thomas Y., Hang’ombe B.M., Moonga L., Mweene A.S., Ogawa H., Nakamura I., Kimura T. (2013). Human parainfluenza virus type 3 in wild nonhuman primates, Zambia. Emerg. Infect. Dis..

[18] Yamaguchi H., Kobayashi S., Ishii A., Ogawa H., Nakamura I., Moonga L., Hang’ombe B.M., Mweene A.S., Thomas Y., Kimura T. (2013). Identification of a novel polyomavirus from vervet monkeys in Zambia. J. Gen. Virol..

[19] Nakayima J., Hayashida K., Nakao R., Ishii A., Ogawa H., Nakamura I., Moonga L., Hang’ombe B.M., Mweene A.S., Thomas Y. (2014). Detection and characterization of zoonotic pathogens of free-ranging non-human primates from Zambia. Parasit. Vectors.

[20] Nakayama E., Yokoyama A., Miyamoto H., Igarashi M., Kishida N., Matsuno K., Marzi A., Feldmann H., Ito K., Saijo M. (2010). Enzyme-linked immunosorbent assay for detection of filovirus species-specific antibodies. Clin. Vaccine Immunol..

[21] Ogawa H., Miyamoto H., Nakayama E., Yoshida R., Nakamura I., Sawa H., Ishii A., Thomas Y., Nakagawa E., Matsuno K. (2015). Seroepidemiological prevalence of multiple species of filoviruses in fruit bats (*Eidolon helvum*) migrating in Africa. J. Infect. Dis..

[22] Ogawa H., Miyamoto H., Ebihara H., Ito K., Morikawa S., Feldmann H., Takada A. (2011). Detection of all known filovirus species by reverse transcription-polymerase chain reaction using a primer set specific for the viral nucleoprotein gene. J. Virol. Methods.

[23] Broadhurst M.J., Brooks T.J., Pollock N.R. (2016). Diagnosis of Ebola virus disease: Past, present, and future. Clin. Microbiol. Rev..

[24] Fisher-Hoch S.P., Perez-Oronoz G.I., Jackson E.L., Hermann L.M., Brown B.G. (1992). Filovirus clearance in non-human primates. Lancet.

[25] CDC Chronology of Marburg Hemorrhagic Fever Outbreaks. http://www.cdc.gov/vhf/marburg/resources/outbreak-table.html.

[26] CDC Chronology of Ebola Hemorrhagic Fever Outbreaks. http://www.cdc.gov/vhf/ebola/resources/outbreak-table.html.

[27] Carroll S.A., Towner J.S., Sealy T.K., McMullan L.K., Khristova M.L., Burt F.J., Swanepoel R., Rollin P.E., Nichol S.T. (2013). Molecular evolution of viruses of the family Filoviridae based on 97 whole-genome sequences. J. Virol..

[28] Changula K., Kajihara M., Mori-Kajihara A., Eto Y., Miyamoto H., Yoshida R., Shigeno A., Hang’ombe B., Qiu Y., Mwizabi D. (2018). Seroprevalence of filovirus infection of Rousettus aegyptiacus bats in Zambia. J. Infect. Dis..

[29] Fisher-Hoch S.P., Brammer T.L., Trappier S.G., Hutwagner L.C., Farrar B.B., Ruo S.L., Brown B.G., Hermann L.M., Perez-Oronoz G.I., Goldsmith C.S. (1992). Pathogenic potential of filoviruses: Role of geographic origin of primate host and virus strain. J. Infect. Dis..

[30] Saj T.L., Sicotte P., Paterson J.D. (2001). The conflict between vervet monkeys and farmers at the forest edge in Entebbe, Uganda. Afr. J. Ecol..

[31] Healy A., Nijman V. (2014). Pets and pests: Vervet monkey intake at a specialist South African rehabilitation centre. Anim. Welf..

[32] Findlay L.H., Hill R.A. (2020). Baboon and vervet monkey crop-foraging behaviors on a commercial South African farm: Preliminary implications for damage mitigation. Hum-Wildl. Interact..

[33] Chomba C., Senzota R., Chabwela H., Mwitwa J., Nyirenda V. (2012). Patterns of human-wildlife conflicts in Zambia, causes, consequences and management responses. J. Ecol. Nat. Environ..

[34] Grobler P., Jacquier M., deNys H., Blair M., Whitten P.L., Turner T.R. (2006). Primate sanctuaries, taxonomy and survival: A case study from South Africa. Ecol. Envir. Anthropol..

[35] Mulangu S., Alfonso V.H., Hoff N.A., Doshi R.H., Mulembakani P., Kisalu N.K., Okitolonda-Wemakoy E., Kebela B.I., Marcus H., Shiloach J. (2018). Serologic evidence of ebolavirus infection in a population with no history of outbreaks in the Democratic Republic of the Congo. J. Infect. Dis..

[36] Steffen I., Lu K., Yamamoto L.K., Hoff N.A., Mulembakani P., Wemakoy E.O., Muyembe-Tamfum J.J., Ndembi N., Brennan C.A., Hackett J. (2019). Serologic prevalence of Ebola virus in Equatorial Africa. Emerg. Infect. Dis..

[37] Steffen I., Lu K., Hoff N.A., Mulembakani P., Okitolonda W.E., Muyembe-Tamfum J.J., Ndembi N., Brennan C.A., Hackett J., Switzer W.M. (2020). Seroreactivity against Marburg or related filoviruses in West and Central Africa. Emerg. Microbes. Infect..

[38] Jain V., Charlett A., Brown C.S. (2020). Meta-analysis of predictive symptoms for Ebola virus disease. PLoS Negl. Trop. Dis..

[39] Kortepeter M.G., Dierberg K., Shenoy E.S., Cieslak T.J. (2020). Medical Countermeasures Working Group of the National Ebola Training and Education Center’s (NETEC) Special Pathogens Research Network (SPRN). Marburg virus disease: A summary for clinicians. Int. J. Infect Dis..

[40] Jaffe K.E., Isbell L.A. (2010). Changes in ranging and agonistic behavior of vervet monkeys (*Cercopithecus aethiops*) after predator-induced group fusion. Am. J. Primatol..

[41] Johnson C., Piel A.K., Forman D., Stewart F.A., King A.J. (2015). The ecological determinants of baboon troop movements at local and continental scales. Mov. Ecol..

[42] Kajihara M., Hang’ombe B.M., Changula K., Harima H., Isono M., Okuya K., Yoshida R., Mori-Kajihara A., Eto Y., Orba Y. (2019). Marburgvirus in Egyptian fruit bats, Zambia. Emerg. Infect. Dis..

[43] Hamilton W.J. (1982). Baboon sleeping site preferences and relationships to primate grouping patterns. Am. J. Primatol..

[44] Randhawa N., Bird B.H., VanWormer E., Sijali Z., Kilonzo C., Msigwa A., Ekiri A.B., Samson A., Epstein J.H., Wolking D.J. (2020). Fruit bats in flight: A look into the movements of the ecologically important Eidolon helvum in Tanzania. One Health Outlook.

[45] Tapanes E., Detwiler K.M., Cords M. (2016). Bat predation by cercopithecus monkeys: Implications for zoonotic disease transmission. Ecohealth.

[46] Monadjem A., Taylor P.J., Cotterill F.P.D., Schoeman M.C. (2010). Bats of Southern and Central Africa. A Biogeographic and Taxonomic Synthesis.

